# Disclosing Influencer Marketing on YouTube to Children: The Moderating Role of Para-Social Relationship

**DOI:** 10.3389/fpsyg.2019.03042

**Published:** 2020-01-21

**Authors:** Sophie C. Boerman, Eva A. van Reijmersdal

**Affiliations:** Amsterdam School of Communication Research, University of Amsterdam, Amsterdam, Netherlands

**Keywords:** YouTube, influencer marketing, children, advertising literacy, para-social relationship, persuasion knowledge, disclosure, brand responses

## Abstract

Watching online videos is becoming an important part of children’s media diets. Children particularly like content that is specifically created for YouTube by YouTube personalities. Because these personalities have a large reach and are considered likeable and credible, they have become social media influencers. For advertisers, these influencers are an interesting channel to reach youth. Therefore, influencers often embed persuasive sponsored messages in their videos to earn money. However, there are concerns about this practice because it is not always clear when a video includes advertising. Therefore, in several countries, guidelines have been developed that state that sponsoring in influencer videos should be disclosed as such. Until now, little is known about the effects of disclosures for influencer videos on children and the boundary conditions for such effects. Therefore, we investigated the effects of a disclosure of sponsored influencer videos on children’s advertising literacy. Additionally, we examined the consequences of the disclosure for children’s responses to the brand, advertised product, and video. We also included the para-social relationship (PSR) that children experience with an influencer as a possible boundary condition for disclosure effects on persuasion. Our experiment amongst children between 8 and 12 years old showed that, when children correctly recalled the disclosure, the disclosure increased their recognition of advertising, and understanding of selling and persuasive intent. Moreover, advertising literacy evoked by the disclosure affected persuasion: The disclosure enhanced brand memory through ad recognition, but also decreased advertised product desire through understanding the selling intent of the video. Furthermore, the PSR of children with the influencer proved to be a boundary condition for disclosure effects on brand attitudes. Only for those children who experienced moderate to low PSRs with the influencer, the disclosure resulted in less positive brand attitudes through understanding selling intent. For children who experienced a strong PSR with the influencer, the understanding that the content had a selling intent did not affect their brand attitudes. These findings show that a disclosure (if noticed and remembered) can be an effective tool to achieve transparency, but also influences the persuasive outcomes of influencer marketing in online videos.

## Introduction

Nowadays, the majority of children between 8 and 11 years old prefers to watch content on YouTube rather than watching TV programs on a TV set (Ofcom, 2018). Children particularly like content that is specifically created for YouTube by YouTube personalities such a vloggers. The YouTube content children consume varies from videos of daily lives (the so-called video blogs or vlogs), to pranks, people playing video games, unboxing products, product reviews, and people showcasing their (musical) talents. The YouTubers who create this content can become very popular and build large communities with occasionally millions of followers and subscribers. Their large network, the popularity of their content, and the fact that children consider YouTubers as likeable, credible, and inspirational characters have made them interesting spokespersons for advertisers (De Jans et al., 2018a; Evans et al., 2018; Folkvord et al., 2019). Hence, YouTubers have become important social media influencers that can reach a young audience.

As influencers, YouTubers are approached by brands to mention, show, or promote a product or brand in their videos in exchange for payment or other reciprocal arrangements (such as free products). For example, Ryan, a hugely popular child YouTuber with 22 million subscribers, unpacks and demonstrates toys from Hasbro in his videos on his channel called Ryan’s world. And, in his videos, Dutch YouTuber Furtjuh bakes cookies using the products of BlueBand, a famous Dutch butter brand.

Influencer marketing on YouTube, also referred to as sponsored content, native advertising, and vlog advertising, raises ethical concerns because this type of advertising is integrated in non-commercial content that is made by an independent content creator, and thus it blurs the lines between what is advertising and what is not (De Jans et al., 2018a; Evans et al., 2018). This subtle and embedded nature makes it difficult for audiences to recognize influencer marketing as advertising (Rozendaal et al., 2011; Buijzen et al., 2010; Boerman et al., 2017; Evans et al., 2018). Children are expected to be even less likely than adults to understand the commercial nature of influencer marketing, because children’s advertising-related knowledge and skills, such as understanding the selling intent of an ad, referred to as *advertising literacy* or *persuasion knowledge*, has often not matured (Rozendaal et al., 2016). Because people need advertising literacy to cope with advertising, this makes children even more susceptible to advertising, and especially to covert, embedded advertising such as influencer marketing. Qualitative research indeed showed that children (9–12 years old) and adolescents (12–16 years old) have difficulties recognizing hidden and embedded advertising in YouTube videos (Martínez and Olsson, 2019; Van Dam and Van Reijmersdal, 2019).

To help both adults and children to recognize influencer marketing, regulators and self-regulatory bodies stress the importance of clearly and conspicuously disclosing influencer marketing, also on YouTube (Federal Trade Commission [FTC], 2017; European Advertising Standards Alliance [EASA], 2018). To our knowledge, only one study exists on the effects of influencer marketing disclosures among children. This study showed that a disclosure can be an effective cue to help children recognize advertising in YouTube videos (De Jans et al., 2018a). Although this finding is promising, there is still a lot to gain in this field. This study aims to add to the literature in three ways.

First, it is important to gain insights into whether influencer marketing disclosures can not only activate children’s recognition of advertising (De Jans et al., 2018a), but also more elaborate and complex components of advertising literacy, such as the understanding that a video has a selling and a persuasive intent. Although ad recognition is an important first step of advertising literacy (John, 1999; Rozendaal et al., 2016), we argue that children need to activate more complex levels of advertising literacy to actually use their cognitive defenses in response to the sponsored content. Importantly, Rozendaal et al. (2009) showed that children’s understanding of persuasive intent reduced their advertised product desire, whereas recognition of advertising and understanding selling intent did not. Thus, our first aim is to investigate to what extent a disclosure [“(YouTuber name) is being paid by (brand) to advertise in his vlog”] can increase three levels of advertising literacy: ad recognition, understanding of selling intent, and understanding of persuasive intent.

Second, the activation of advertising literacy stimulated by a disclosure may influence children’s cognitive and affective responses to the brand, product, and video. Cognitively, because the activation of advertising literacy requires systematic processing of the sponsored content, a disclosure may (indirectly) increase brand recall. When looking at the affective side, influencers are seen as personal, credible, and easy to relate to sources (De Veirman et al., 2017). Children consider YouTubers as useful sources of information on which products to buy and how to use these products (Martínez and Olsson, 2019). Likewise, research has shown that influencers can positively influence brand attitudes and purchase intentions (Jin and Phua, 2014; Lee and Watkins, 2016; Djafarova and Rushworth, 2017; Domingues Aguiar and Van Reijmersdal, 2018). However, when a disclosure does help children to use their advertising literacy in response to a sponsored YouTube video, this may also lead to reactance (Van Reijmersdal et al., 2017; Van Dam and Van Reijmersdal, 2019). Prior research has indeed indicated that a disclosure on YouTube activated children’s affective advertising literacy (a general disliking of advertising in vlogs) and this consequently led to lower influencer trustworthiness and ultimately decreased purchase intentions (De Jans et al., 2018a). To add to these findings, the second aim of this study is to investigate whether a disclosure may take away the advantages of influencer marketing, and decrease other persuasive outcomes, namely children’s brand attitude, advertised product desire, and video attitude.

Third, we aim to gain insight into the boundary conditions of disclosure effects by focusing on whether its consequences are a function of the level of the para-social relationship (PSR) that children experience with the YouTuber. Previous research showed that people can develop long-lasting relationships with influencers on YouTube (Lee and Watkins, 2016; Munnukka et al., 2019). We propose that the negative affective consequences of the activation of advertising literacy may be contingent upon the PSR children experience with an influencer. When children have developed a strong PSR with an influencer, they may be less critical toward him/her and the content that he/she creates, and thus not feel reactant when they understand that the influencer was paid to show or mention a brand in a video. Therefore, we expect that the activation of advertising literacy does not negatively affect the brand, product, and video responses for children that feel a strong PSR with the influencer.

In this study, we focus on children aged 8–12. The literature shows that children of this age are able to understand the intent of advertising (John, 1999; Rozendaal et al., 2009). A disclosure can only activate this knowledge, when it is present in children. Moreover, children in this age category are considered “cued processors” which means they need external cues to activate their existing knowledge structures (John, 1999). A disclosure can serve as an external cue that can help children of this age to activate their advertising literacy.

### Sponsorship Disclosures and Advertising Literacy

The main aim of disclosures is to make people aware of the persuasive nature of influencer marketing (Bladow, 2017). Because influencer marketing is often masked as a non-commercial post-created by the influencer itself, people are assumed to need a disclosure to activate their advertising literacy when confronted with this type of content (Hellemans et al., 2015; Bladow, 2017).

Advertising literacy comprises several components of which recognition of content as being a form of advertising and understanding of the selling intent and of the persuasive intent of the content are most often examined in disclosure studies (Boerman and Van Reijmersdal, 2016). Recognition of influencer marketing as being advertising (ad recognition) is defined as the ability to “differentiate sponsored content from other media content” (Boerman et al., 2018, p. 674). Understanding the selling intent of influencer marketing refers to “understanding that the aim of the content is to sell products” (Boerman et al., 2018, p. 674), whereas understanding persuasive intent is “the understanding that the aim of the content is to influence consumers’ behavior by changing their mental states, for example their attitudes and cognitions about a product” (Boerman et al., 2018, p. 674).

Research among adults has shown that disclosures of embedded advertising, including influencer marketing, can activate adults’ ad recognition and their understanding of the selling and persuasive intent of the content (e.g., Van Reijmersdal et al., 2015a; Boerman et al., 2017; Evans et al., 2017; An et al., 2018; De Veirman and Hudders, 2019). These effects can be explained by the Persuasion Knowledge Model (Friestad and Wright, 1994). Disclosures provide information about the commercial or persuasive character of embedded advertising and as such may alert people to the fact that the content is advertising and not just entertainment or information (Boerman and Van Reijmersdal, 2016). As such, disclosures can activate people’s advertising literacy, which would not happen without a disclosure, due to the fact that the advertising is masked as non-commercial content (Bladow, 2017). The disclosure can help people to think of advertising and related constructs and thus triggers a process of activating existing knowledge structures that are related to advertising and persuasion (Friestad and Wright, 1994).

The question remains whether influencer marketing disclosures can activate *children’s* advertising literacy. First, children have less mature levels of advertising literacy than adults (Rozendaal et al., 2010; Verhellen et al., 2014). If advertising literacy is not fully-developed, disclosures may not be able to activate this knowledge. Second, influencers offer highly entertaining, involving, and attention-grabbing content, which is likely to absorb children’s cognitive resources (Buijzen et al., 2010). This leaves little room for children to activate their advertising literacy. Even though a disclosure is present, it may not activate children’s advertising literacy because they are occupied by the entertaining content. Thus, the entertaining nature of this form of embedded advertising, may make it very hard for children to “stop and think” about its persuasive nature (Rozendaal et al., 2011; Hudders et al., 2017).

The empirical evidence on the effects of disclosures on activating children’s advertising literacy is limited and mixed. With respect to ad recognition, some studies showed positive effects of disclosures on the activation of this type of advertising literacy (De Jans et al., 2018a, b; Coates et al., 2019). More specifically, Coates et al. (2019) and De Jans et al. (2018a) showed that exposure to a disclosure in an influencer marketing video led to higher recognition of the content as being advertising than no disclosure among children. With respect to the activation of children’s understanding of the selling and persuasive intent of embedded advertising, some studies found no effects (An and Stern, 2011; Panic et al., 2013), whereas another did find effects on a combined measure of advertising literacy including both ad recognition and understanding intent (De Pauw et al., 2018). One reason for these mixed findings could be children’s attention to and recall of the disclosure. Research amongst adults and adolescents has shown that disclosure effects on the activation and use of persuasion knowledge often only occurs when people notice the disclosure (e.g., Boerman et al., 2014, 2017; Van Reijmersdal et al., 2017).

Overall, we propose that a disclosure can activate all three elements of advertising literacy. This leads to our first hypothesis:

H1: A sponsorship disclosure (vs. no disclosure) increases (a) the recognition of the YouTube video as advertising, (b) the understanding of the selling intent of the video, and (c) the understanding of the persuasive intent of the video.

### Consequences of Disclosures for Responses to the Brand, Product, and Video

It is widely assumed that cognitive advertising defenses – such as ad recognition, understanding selling intent, and understanding persuasive intent – reduce children’s susceptibility to advertising effects (Rozendaal et al., 2011). Children need to acquire advertising literacy to cope with advertising. If they do not have this knowledge and cannot use this in response to an ad, they are susceptible to its effects, which could lead to undesired consequences (Rozendaal et al., 2011; Mizerski et al., 2017). We expect that the activation of advertising literacy, stimulated by a disclosure, has various consequences based on different processes: a cognitive process that may enhance brand recall and an affective process that influences brand attitude, advertised product desire, and video attitudes.

With regard to the cognitive process, we propose that the disclosure enhances the level of cognitive elaboration of the sponsored content and consequently increases brand recall. Buijzen et al. (2010) introduced the investigative framework for young people’s processing of commercial media content (PCMC). In their PCMC model, the authors describe that applying advertising literacy to a message requires critical systematic processing of the content (Buijzen et al., 2010). This means that when the disclosure is able to activate children’s advertising literacy, the application of the awareness and understanding of the advertising in the video requires cognitive systematic processing of the advertising. Systematic processing is characterized by high levels of explicit recall of the persuasive message and the advertised product or brand (Buijzen et al., 2010).

Eye tracking research into the effects of disclosures of brand placement in television programs showed that a disclosure can induce more systematic processing as a disclosure increases the visual attention people pay to the brand in the video (Boerman et al., 2014; Smink et al., 2017; Guo et al., 2018). This increased attention explains the positive effects that have been found of disclosures on brand recall amongst adolescents (13–17 years old; Van Reijmersdal et al., 2017) and adults (e.g., Boerman et al., 2012, 2014; Guo et al., 2018). Therefore, we expect a cognitive process in which the disclosure primes the advertising, which activates ad literacy, and thus leads to more systematic processing of the sponsored content and increased brand recall.

Next to this cognitive process, a disclosure and the activation of advertising literacy may also influence children’s affective responses toward the brand, product, and video. The activation of advertising literacy enables children to use their knowledge and skills to cope with the sponsored content. Scholars have argued that rather than using cognitive defenses, children often use affective advertising literacy as a defense against advertising under conditions of low elaboration (Rozendaal et al., 2011). One reason for this is that children often do not stop and think about the advertisement in some considerable depth. However, we argue that a disclosure may change a low elaboration situation into a more systematic one.

A disclosure should be a helpful cue for children to stop and think about the persuasive nature of a YouTube video. We argue that children may defend themselves against persuasion when they are able to apply more complex knowledge, such as the understanding of the video’s selling and persuasive intent. When a disclosure does make children “stop and think,” and enables them to critically evaluate not just whether the video contains advertising, but also its persuasive and selling intent, this may trigger feelings of psychological reactance (Van Reijmersdal et al., 2017; Van Dam and Van Reijmersdal, 2019). People want to have control over their choices and thus are likely to resist threats to this freedom (Brehm, 1966; Fransen and Fennis, 2014). Thus, when a disclosure activates advertising literacy, this may motivate children to resist the persuasion attempt. In the case of YouTube influencer marketing, this reactance may result in more negative attitudes toward the advertised brand and less desire for the product in the video. In addition, understanding that the video was not purely made for fun or entertainment, but also had a commercial purpose, may instigate a “change of meaning” (Friestad and Wright, 1994). Children do not seem to like advertising in YouTube videos (Folkvord et al., 2019), especially when they doubt the honesty of the influencer and realize that a video actually has a commercial purpose (Van Dam and Van Reijmersdal, 2019). The realization that a video is actually advertising may thus alter their response to the video. We argue that children become more skeptical toward the content itself, which leads to more negative attitudes toward the video.

The literature found mixed results with regard to the effects of disclosures and advertising literacy on persuasion. With respect to brand attitude, studies amongst children showed that a disclosure can have a negative effect on brand preference in advergames (An and Stern, 2011), whereas others found no effect of a disclosure in TV programs on brand attitude (Van Reijmersdal et al., 2017). In addition, De Pauw et al. (2018) found that a disclosure can lead to more positive brand attitudes among children who are less skeptical toward brand placement. The same inconsistent findings are found for disclosures studies amongst adults (Boerman and Van Reijmersdal, 2016). The empirical evidence for the relationship between advertising literacy and brand attitude is also inconclusive among children. Most studies fail to find an effect of advertising literacy on brand attitude (e.g., Mallinckrodt and Mizerski, 2007; Van Reijmersdal et al., 2012; Mizerski et al., 2017), but some exceptions do find a negative effect (e.g., Waiguny et al., 2012; for a review see Mizerski et al., 2017).

Furthermore, with regard to product desire, prior studies found a negative effect of a disclosure in an advergame on children’s intention to purchase the advertised product (Panic et al., 2013), and a negative indirect effect of a disclosure on purchase intentions via the activation of affective advertising literacy (a general disliking of advertising in vlogs) and perceived trustworthiness of the influencer (De Jans et al., 2018a). This is in line with research indicating that children’s understanding of persuasive intent reduces advertised product desire (Rozendaal et al., 2009). In addition, this negative disclosure effect on purchase intention was also found among adults in the context of brand placement in movies (Tessitore and Geuens, 2013). However, there is also evidence of the opposite. In the context of influencer marketing for unhealthy food, research found that a disclosure made children eat *more* of the advertised product and more of the provided snacks in general (Folkvord et al., 2017; Coates et al., 2019). Additionally, studies have found positive effects of advertising literacy on purchase intentions or advertised product desired (e.g., Rozendaal et al., 2009; Van Reijmersdal et al., 2015b; Vanwesenbeeck et al., 2017), while others have found no evidence for the relationship between advertising literacy and product desire (Mallinckrodt and Mizerski, 2007).

Finally, to our knowledge, there are no studies that considered the effects of a disclosure on children’s attitude toward the sponsored content itself (such as the video). Prior research has failed to find evidence for a link between advertising literacy and attitude toward an advergame among children (Van Reijmersdal et al., 2012). However, research among adults found that a disclosure decreases (Hwang and Jeong, 2016; Boerman et al., 2017) or does not influence (Sweetser et al., 2016; Krouwer et al., 2017) people’s evaluation of the sponsored content. Based on these findings, reactance theory and the anticipated feeling of being deceived by the influencer, we propose that a disclosure and the consequent activation of advertising literacy may lower video attitudes.

In sum, we propose that a disclosure instigates a cognitive mechanism, in which children process the commercial content more systematically, and thus improves brand recall. Furthermore, a disclosure is assumed to make children less susceptible to advertising, because it helps them to recognize advertising and understand its selling and persuasive intent. When children are less susceptible to influencer marketing, this means that they are able to critically asses the advertisement and cope with the persuasion attempt. Despite the inconclusive empirical evidence, we propose that the activation of these three levels of advertising literacy negatively influence children’s brand attitude, advertised product desire, and video attitude:

H2: A sponsorship disclosure (vs. no disclosure) increases (a) brand recall, and decreases (b) brand attitudes, (c) advertised product desire, and (d) video attitudes, through the activation of the three components of advertising literacy.

### Moderating Role of PSR

When children repeatedly watch content made by the same YouTubers, they can become fans and even develop a PSR with specific YouTubers. A PSR is the illusion of having an enduring, personal relationship with a media personality (Horton and Wohl, 1956; Tsai and Men, 2013). It takes time to develop PSR because it requires an individual to interact with the person, to get to know him or her, develop attitudes toward the person, and to experience feelings of intimacy. Although this happens via media, this long-term contact resembles the development of a real relationship (Russell et al., 2006).

PSR is often used interchangeably with the notion of para-social *interaction* (PSI). However, where PSI refers to the perception of the media personality as an intimidate conversational partner that usually arises during the interaction, PSR refers to an enduring relationship that encompasses more than one interaction or exposure (Dibble et al., 2016; Munnukka et al., 2019).

Social media, such as YouTube, are the perfect place for developing PSR and PSI. Via social media, followers have the opportunity to interact directly with that person and to continually expose themselves to details of a person’s life (Colliander and Dahlén, 2011). Through continuous interactions with social influencers, they can become part of the daily life of followers and create a feeling of intimacy, similarity, and closeness (Lee and Watkins, 2016; Chen, 2018; Munnukka et al., 2019). Previous studies have found evidence for experiences of PSR and PSI with influencers on YouTube (Lee and Watkins, 2016; Folkvord et al., 2019; Munnukka et al., 2019), on Facebook (Tsai and Men, 2013), on Instagram (Chen, 2018), and with bloggers (Colliander and Dahlén, 2011).

The extent to which people perceive PSI with a YouTube influencer seems to depend upon the perceived social and physical attractiveness of this influencer (Lee and Watkins, 2016). Moreover, research has shown that a disclosure (“Contains advertising!”) in a YouTube video can indirectly influence PSI, by increasing children’s disliking of the advertising in the video which consequently reduced the perceived trustworthiness of the influencer, which then decreases PSI (De Jans et al., 2018a). This suggests that children’s critical assessment of advertising in a YouTuber’s video can damage the PSI they feel with this influencer during that video. However, these studies both focused on PSI and not necessarily on the long-term relationship (i.e., PSR) children perceive with a YouTube influencer.

Because PSR takes time to develop and does not depend on one single exposure, we expect that the extent to which children experience PSR with a YouTuber may not be influenced by a disclosure in one video. Alternatively, we expect that the extent to which children perceive PSR with an influencer may provide a boundary condition that explains why some children respond more negatively to advertising in YouTube video than others. Thus, we propose that PSR moderates the effect of advertising literacy on brand, product, and video responses.

More specifically, we expect that the negative effect on brand attitude, advertised product desire, and video attitude does not appear for children with a strong PSR with the influencer. This is based upon the notion that PSR has been shown to increase the perceived credibility of a YouTube influencer (Munnukka et al., 2019). Therefore, children who feel a strong PSR with the YouTuber are more likely to perceive this influencer to be credible. In addition, they may be less critical toward the influencer when they feel connected and similar to them. Thus, when children have developed a strong PSR with an influencer, they may be less critical toward him/her and the content that he/she creates. This would mean that children with a strong PSR may not feel reactant when they understand that the influencer was paid to show or mention a brand in a video. Consequently, we expect that there is no negative effect of ad literacy on brand, product, and video responses for children with high levels of PSR. In line with this reasoning, Dekker and Van Reijmersdal (2013) found that a disclosure in a sponsored episode of The Oprah Winfrey Show did not affect the acceptance of the claims Oprah made about the advertised product for people who believed Oprah to be highly credible. The disclosure did have a negative effect on product claim acceptance for those who did not find Oprah credible.

**FIGURE 1:**
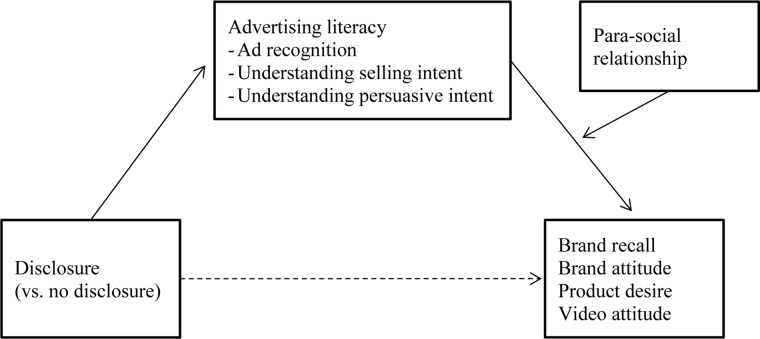
Conceptual model.

In sum, we propose that the level of PSR that children experience with an influencer is a contingent moderator (Holbert and Park, 2019) of the negative effect that the disclosure and activation of advertising literacy have on children’s brand, product, and video responses. This leads to the following moderated mediation hypothesis, which is also depicted in Figure 1:

H3: The effect of the disclosure via the three components of advertising literacy on (a) brand attitudes, (b) advertised product desire, and (c) video attitudes is negative and statistically significant for children with low levels of PSR and non-significant for children with a strong PSR with the YouTuber.

## Materials and Methods

### Design and Sample

To test our hypotheses, we conducted an experiment with a one factorial (sponsorship disclosure vs. no sponsorship disclosure) between subjects design. We collected data at three different schools. The school were located in different cities in the Netherlands (Amsterdam *n* = 23, Hilversum *n* = 30, and Rotterdam *n* = 59). In total, 118 children participated in the study, of which 112 completed it. These 112 children were between 8 and 12 years old (*M* = 10.43, *SD* = 0.89; 50% was 11 years old), divided over three primary school grades, and most of them were boys (56.3%). Participants were randomly assigned to one of the two conditions (disclosure condition *n* = 54, no disclosure condition *n* = 58).

### Procedure

The study was approved by the ERB of the University of Amsterdam. Parents were sent an email informing them about the study in advance, asking for their passive consent. If they did not want their child to participate in the study, they had the opportunity to inform the researchers or schools beforehand. None of the parents objected to their child’s participation.

The experiment took place within a designated computer room, or in the classroom on laptops. The children were told that they were participating in a study into vlogs conducted by researchers from the University of Amsterdam. The children were asked to watch the video and were asked to answer some questions about the video and the YouTuber afterward. They were assured that there were no right or wrong questions and that they could ask the researcher questions when anything was unclear. They were also allowed to stop participating whenever they wanted (none of them did).

All children used an individual set of headphones to watch and listen to the video individually. The questionnaire allowed children to continue after the video had finished. The questionnaire started with questions about their video attitude, followed by their familiarity with the YouTuber, the frequency of watching his videos, and PSR. It continued with brand recall, brand familiarity, ad recognition, understanding of selling intent, understanding of persuasive intent, brand attitude, and advertised product desire. It ended with a manipulation check and several control variables (frequency of watching videos on YouTube, frequency of eating the brand’s products, sex, age, and grade). The children were then thanked and the researcher debriefed them.

### Stimulus Materials

We used an existing vlog of a popular YouTuber with more than two million followers. We edited and shortened the 26 min original vlog into a vlog that lasted three and a half minutes. In this vlog, the YouTuber tells that he wants to start eating more healthily and therefore is going to have fish sticks for dinner. He shows his lunch, has a picknick with his girlfriend, and then prepares the fish sticks with his girlfriend. They talk about fish sticks quite elaborately, for instance by repeatedly saying that fish sticks are healthy and that they used to eat them as a child, and the YouTuber tells a story about the actors that play in the commercials for the brand. The YouTuber mentions the brand name three times and shows the product packaging with the brand name on it once for 4 s.

In the disclosure condition, the video was preceded by a sponsorship disclosure. This disclosure was shown in large white letters on a black background for 10 s and said: “(YouTuber name) is being paid by (brand) to advertise in his vlog.” This disclosure was based upon and in line with the FTC’s guides concerning the use of endorsements and testimonials in advertising (Federal Trade Commission [FTC], 2017). Following prior research, the disclosure clearly and directly conveys the paid relationship between the producer and the sponsor (Hyman et al., 2017; Wojdynski et al., 2017). In the no disclosure condition, the disclosure was not shown and the video started immediately.

### Measures

We measured the three levels of advertising literacy by applying the general scales developed by Rozendaal et al. (2016) to this video and brand. The items were all measured with six-point scales (1 = *No, certainly not*, 2 = *No, I do not think so*, 3 = *No, maybe*, 4 = *Yes, maybe*, 5 = *Yes, I think so*, 6 = *Yes, certainly*).

#### Ad Recognition

We measured recognition of the sponsored video as being advertising with two questions: “Was there advertising for (Brand) fish sticks in the video?” and “Was the video sponsored by (Brand) fish sticks?” Mean scores were calculated to create a single measure of ad recognition (Spearman–Brown = 0.66, *M* = 4.11, *SD* = 1.39).

#### Understanding of Selling Intent

Children’s understanding of the selling intent of the video was measured by asking them three questions: “Was the video made to make children ask their parents to buy (Brand) fish sticks?” “Was the video made to make people buy (Brand) fish sticks?” and “Was the video made to make you buy (Brand) fish sticks with your own pocket money?” The mean score of the three items was used as a measure of understanding of selling intent (*M* = 3.08, *SD* = 1.23; α = 0.80).

#### Understanding of Persuasive Intent

To gain insights into children’s understand of the persuasive intent of the video, we asked them three questions: “Was the video made to make people like (Brand) fish sticks?” “Was the video made to make people want to have (Brand) fish sticks?” and “Was the video made to make people think positively about (Brand) fish sticks?” The mean score of the three items was used as a measure of understanding of persuasive intent (*M* = 3.65, *SD* = 1.22; α = 0.79).

#### Brand Attitude

We measured brand attitude with four questions: “Do you think (Brand) fish sticks is…” followed by two positive and two negative adjectives: “nice,” “tasty,” “stupid,” and “disgusting” (Van Reijmersdal et al., 2012, 2017). The scale anchors were adjusted to the questions (e.g., 1 = *totally not tasty* to 6 = *very tasty*). The two negative items were recoded, and the mean scores were used as a measure of brand attitude with high scores corresponding to positive attitudes (eigenvalue = 3.19, explained variance = 79.63%, α = 0.91; *M* = 4.43, *SD* = 1.28).

#### Advertised Product Desire

Children’s desire for the advertised product was measured by asking them “Would you like to have (Brand) fish sticks for dinner today?” (based on Rozendaal et al., 2009); 1 = *No, certainly not*, 6 = *Yes, definitely*; *M* = 3.42, *SD* = 1.81).

#### Video Attitude

The measure of video attitude was similar to the brand attitude measure. We asked the participants four questions: “Did you think the video was…” followed by “nice,” “funny,” “stupid,” and “boring” (Derbaix and Pecheux, 2003; D’Alessio et al., 2009; Van Reijmersdal et al., 2012). The scale anchors were adjusted to the questions (e.g., 1 = *totally not funny* to 6 = *very funny*). The negative items were recoded, and the mean scores were used as a measure of video attitude with high scores corresponding to positive attitudes (eigenvalue = 2.51, explained variance = 62.62%, α = 0.79; *M* = 3.72, *SD* = 0.92).

#### Para-Social Relationship (PSR)

We measured children’s PSR with the YouTuber with six questions: “Do you want to do the same things as Enzo Knol?” “Do you want to be similar to Enzo Knol?” “Do you want to be like Enzo Knol?” “Do you want to meet Enzo Knol in person?” “Would you be sorry if Enzo Knol would stop vlogging?” and “Do you find it annoying when Enzo Knol makes a mistake?” (1 = *No, certainly not*, 2 = *No, I do not think so*, 3 = *No, maybe*, 4 = *Yes, maybe*, 5 = *Yes, I think so*, 6 = *Yes, certainly*; based on scales used by Rubin and Perse, 1987; Hoffner, 1996; Auter and Palmgreen, 2000; Russell et al., 2006). Factor analysis revealed that the last question (“Do you find it annoying when Enzo Knol makes a mistake?”) did not load onto the factor (component loading = −0.10) and that the reliability would improve by excluding it (α changed from 0.76 to 0.84 when deleted). Therefore, we decided to use the mean score of the first five questions as a measure of PSR (eigenvalue = 3.11, explained variance = 62.22%; α = 0.84; *M* = 2.70, *SD* = 1.18).

#### Manipulation Check

As a manipulation check, we asked the participants: “Did you see a white text on a black screen at the beginning of the video?” (1 = *No*, 2 = *Yes*). If a child answered yes, we asked to fill out what the text said. Answers were coded as correct (when description of the disclosure included something about advertising, promotion, or the brand) or incorrect.

Correct recall of the disclosure was significantly different between the two conditions, χ^2^(1) = 28.33, *p* < 0.001. Most of the 58 children in the disclosure condition (79.3%) did recall seeing the black screen with the white text at the beginning, and 48.3% correctly recalled what the disclosure said. A majority of the children in the no disclosure condition (96.3%) correctly indicated not to have seen a disclosure or filled out incorrect information about the text they supposedly saw. Disclosure recall was not related to participant’s age, χ^2^(4) = 3.93, *p* = 0.415.

Because prior studies have shown that disclosure recall is often a prerequisite for disclosure effects (e.g., Boerman et al., 2014; Van Reijmersdal et al., 2017), we decided to take disclosure recall into account in our analyses. To do so, we created three quasi-experimental conditions: the no disclosure condition (*n* = 50, excluding the two participants who incorrectly indicated to have seen a disclosure), the group who was exposed to the disclosure but did not correctly recall it (*n* = 30), and the group of participants who correctly recalled the disclosure (*n* = 28). All analyses were done with these three groups unless stated otherwise.

#### Control Variables

We asked the participants whether they were familiar with the YouTuber and the brand (1 = *No*, 2 = *Yes*), how often they watched his videos, how often they watched videos on YouTube, and how often they eat (Brand) fish sticks (1 = *Never*, 2 = *Rarely*, 3 = *Sometimes*, 4 = *Often*, 5 = *Almost every day*, 6 = *Every day*). Most children knew the YouTuber (95.5% said yes), but the majority (59.8%) said never or almost never to watch his videos, while some (9.9%) watched his videos daily or almost daily. Most children (75.9%) did watch YouTube videos often to daily (scores 4–6). Additionally, 82.1% of the children knew the brand (Brand). More than half of the children (58.0%) said to never or rarely eat (Brand) fish sticks, whereas 37.5% said to eat them sometimes.

## Results

### Randomization Check

The three experimental groups did not differ with respect to sex, χ^2^(2) = 1.14, *p* = 0.566, age, *F*(2, 107) = 2.17, *p* = 0.119, familiarity with the YouTuber, χ^2^(2) = 3.38, *p* = 0.184, frequency of watching vlogs by the YouTuber, *F*(2, 107) = 0.89, *p* = 0.413, frequency of watching YouTube videos, *F*(2, 107) = 0.28, *p* = 0.754, brand familiarity, χ^2^(2) = 2.53, *p* = 0.282, and frequency of eating the product, *F*(2, 107) = 0.44, *p* = 0.643. The experimental groups were also evenly distributed amongst the three schools, χ^2^(4) = 3.14, *p* = 0.535, and the three grades, χ^2^(4) = 6.94, *p* = 0.139. Because age plays an important role in children’s advertising literacy between the ages of 8 and 12 (John, 1999; Rozendaal et al., 2011) we decided to control for age in all analyses by including it as a covariate^1^.

### Effect of Disclosure on the Activation of Ad Literacy

To test the first hypothesis, we did an MANCOVA with the three disclosure conditions as factor; ad recognition, understanding of selling intent, and understanding persuasive intent as dependent variables; and age as covariate. The results showed a significant effect of the disclosure on the three dimensions of advertising literacy, Pillai’s Trace = 0.27, *F*(6, 204) = 5.27, *p* < 0.001. Table 1 presents an overview of the mean scores of the dependent variables in the three disclosure conditions.

**TABLE 1:** Effect of disclosure (vs. no disclosure) on advertising literacy and brand, product, and video responses.

	No disclosure (*n* = 50)	Disclosure not recalled (*n* = 30)	Disclosure recalled (*n* = 28)
Ad recognition	3.55^(1.28)a^	4.08^(1.43)a^	5.13^(0.95)b^
Understanding of selling intent	2.66^(1.13)a^	2.94^(1.20)a^	3.92^(1.00)b^
Understanding persuasive intent	3.31^(1.20)a^	3.44^(1.24)a^	4.46^(0.88)b^
Brand recall	55.8%^a^	66.7%^a^	92.9%^b^
Brand attitude	4.45^(1.12)a^	4.30^(1.57)a^	4.46^(1.30)a^
Advertised product desire	3.54^(1.77)a^	3.23^(1.89)a^	3.39^(1.91)a^
Video attitude	3.74^(0.94)a^	3.70^(0.91)a^	3.63^(0.89)a^

**^*a,b*^Means with a different superscript in the same row differ significantly at p < 0.05.**

The disclosure conditions had a significant effect on children’s ad recognition, *F*(2, 103) = 14.64, *p* < 0.001, understanding of the selling intent, *F*(2, 103) = 11.12, *p* < 0.001, and understanding of the persuasive intent of the video, *F*(2, 103) = 9.13, *p* < 0.001.

Bonferroni *post hoc* analyses showed that, when children correctly recalled the disclosure (*M* = 5.13, *SD* = 0.95) it significantly increased their recognition of advertising in the video, compared to no disclosure (*M* = 3.55, *SD* = 1.28, *p* < 0.001), and compared to when the disclosure was not recalled (*M* = 4.08, *SD* = 1.43, *p* = 0.005).

For those children who recalled seeing the disclosure, it also improved their understanding of the selling intent (*M* = 3.92, *SD* = 1.00), compared to children who were not exposed to a disclosure (*M* = 2.66, *SD* = 1.13, *p* < 0.001) and to children who did not recall the disclosure (*M* = 2.94, *SD* = 1.20, *p* = 0.004).

Additionally, the recalled disclosure (*M* = 4.46, *SD* = 0.88) increased children’s understanding of the persuasive intent of the video, compared to no disclosure (*M* = 3.31, *SD* = 1.20, *p* < 0.001) and the non-recalled disclosure (*M* = 3.44, *SD* = 1.24, *p* = 0.003).

For all three measures of ad literacy, the disclosure not recalled condition did not significantly differ from the no disclosure condition (*p*’s > 0.185).

This means that we find support for H1: A sponsorship disclosure increases children’s ad recognition, understanding of selling intent, and understanding of persuasive intent. However, this effect only occurs when children correctly recalled seeing this disclosure. When they did not notice or incorrectly remembered its content, the disclosure had no effect.

### Indirect Effects on Brand, Product, and Video Responses

To test H2, we ran a mediation model for each dependent variable (i.e., brand recall, brand attitude, advertised product desire, and video attitude) using Model 4 and 10,000 bootstrap samples in PROCESS version 3.3 (Hayes, 2017). In these models, we included the three disclosure conditions as multicategorical independent variable and used the indicator coding system to compare the no disclosure condition to the disclosure recalled and the disclosure not recalled groups. Furthermore, ad recognition, understanding of selling intent, and understanding of persuasive intent were included as parallel mediators; and age as covariate. Table 2 presents the outcomes of the mediation analyses for the recalled disclosure (vs. no disclosure).

**TABLE 2:** Mediation effects of the recalled disclosure (vs. no disclosure) on brand, product, and video responses via advertising literacy.

	Disclosure > Mediator	Mediator > DV	Total effect	Direct effect	Indirect effect
Brand recall				1.72 (0.90)	
Ad recognition	**1.54 (0.14)^∗∗∗^**	**0.59 (0.24)^∗^**			**0.91 (0.50) CI [0.19, 2.14]**
Understanding selling intent	**1.24 (0.26)^∗∗∗^**	0.15 (0.36)			0.18 (0.63) CI [−0.77, 1.72]
Understanding persuasive intent	**1.13 (0.27)^∗∗∗^**	−0.26 (0.36)			−0.30 (0.58) CI [−1.75, 0.51]
Brand attitude			−0.07 (0.30)	−0.10 (0.34)	
Ad recognition		0.15 (0.11)			0.22 (0.19) CI [−0.15, 0.59]
Understanding selling intent		−**0.36 (0.17)^∗^**			−0.44 (0.27) CI [−1.00, 0.08]
Understanding persuasive intent		0.22 (0.17)			0.25 (0.23) CI [−0.20, 0.72]
Product desire			−0.27 (0.42)	−0.15 (0.48)	
Ad recognition		0.05 (0.16)			0.07 (0.24) CI [−0.40, 0.56]
Understanding selling intent		−**0.56 (0.24)^∗^**			−**0.70 (0.35) CI [**−**1.47,** −**0.10]**
Understanding persuasive intent		0.45 (0.24)			0.51 (0.30) CI [−0.04, 1.15]
Video attitude			−0.11 (0.22)	−0.08 (0.25)	
Ad recognition		0.05 (0.08)			0.08 (0.13) CI [−0.16, 0.35]
Understanding selling intent		−0.02 (0.13)			−0.03 (0.17) CI [−0.35, 0.33]
Understanding persuasive intent		−0.07 (0.13)			−0.08 (0.15) CI [−0.41, 0.19]

*Significant effects are bold. DV, dependent variable; CI, 95% bootstrap confidence intervals. ^∗∗∗^p < 0.001, ^∗^p < 0.05.
*

The mediation analyses again show the significant effects of the recalled disclosure on the three components of advertising literacy: ad recognition (*b* = 1.54, *SE* = 0.14, *p* < 0.001), understanding selling intent (*b* = 1.24, *SE* = 0.26, *p* < 0.001), and understanding of persuasive intent (*b* = 1.13, *SE* = 0.27, *p* < 0.001).

With regard to **brand recall**, the mediation analyses showed a marginally significant direct effect of the disclosure on brand recall (*b* = 1.72, *SE* = 0.90, *p* = 0.056). Because the PROCESS macro does not provide a total effect for dichotomous dependent variable, we ran a logistic regression with two dummy variables representing the disclosure conditions (disclosure recalled and disclosure not recalled, making the no disclosure condition the reference) and age as independent variables, and brand recall as dependent variable. The results showed that the recalled disclosure had a significant positive total effect on brand recall (*b* = 2.42, *SE* = 0.84, *odds ratio* = 11.30, *p* = 0.004). Children who correctly recalled the disclosure in the video were more likely to recall the brand (92.9%) compared to those who were not exposed to the disclosure (55.8%).

Furthermore, the mediation analyses showed that this effect was mediated by the recognition of the vlog as advertising, indirect effect = 0.91, boot *SE* = 0.50, 95% CI [0.19, 2.14]. Thus, the recalled disclosure increased ad recognition, which consequently increased brand recall. We found no significant indirect effects via understanding of selling intent and understanding of persuasive intent. We also did not find any significant effects of the disclosure for the children who did not correctly recall it (vs. no disclosure).

With respect to **brand attitude**, we found no significant total or direct effect of the disclosure conditions (vs. no disclosure), and no indirect effects via the three components of ad literacy.

For **advertised product desire**, the results showed a significant indirect effect of the recalled disclosure (vs. no disclosure) via understanding of selling intent, indirect effect = −0.70, boot *SE* = 0.35, 95% CI [−1.47, −0.10]. The recalled disclosure increased the understanding of the selling intent of the video, which in turn decreased advertised product desire (*b* = −0.56, *SE* = 0.24, *p* = 0.023). We found no indirect effects on advertised product desire via ad recognition and understanding of persuasive intent.

Lastly, we found no significant total, direct effect, or indirect effects on **video attitude**.

Overall, our results partially support H2a: The disclosure had a positive effect on brand recall via one of the advertising literacy components (i.e., ad recognition). We also found partial support for H2c: The disclosure had a significant negative effect on advertised product desire via one of the advertising literacy components (i.e., understanding of selling intent). Again, these conclusions can only be drawn for those children who correctly recalled the disclosure. We did not find support for H2b and H2d.

### Moderating Effect of PSR

To test the moderating effect of PSR, we used model 14 in PROCESS version 3.3 (Hayes, 2017). We ran separate analyses for the three dependent variables (i.e., brand attitude, advertised product desire, and video attitude). In these models, we included the indicator coded disclosure variable as independent variable; ad recognition, understanding of persuasive intent, and understanding of selling intent as parallel mediators; age as covariate; and PSR as moderator of the effect of the advertising literacy components on the dependent variables.

With respect to **brand attitude**, we found a significant index of moderated mediation for the effect of the recalled disclosure (vs. no disclosure) on brand attitude via the understanding of selling intent, index = 0.38, boot *SE* = 0.20, 95% CI [0.01, 0.79]. This suggests that there is a conditional indirect effect of the disclosure on brand attitude via understanding of selling intent. The negative indirect effect is strongest and significant for those with low levels of PSR (16th percentile, PSR = 1.55), indirect effect = −0.99, boot *SE* = 0.27, 95% CI [−1.72, −0.22]. The indirect effect is weaker but significant for the children with moderate levels of PSR (50th percentile, PSR = 2.50), indirect effect = −0.63, boot *SE* = 0.27, 95% CI [−1.16, −0.10], and is not significant for children who experienced relatively high PSR (84th percentile, PSR = 4.00), indirect effect = −0.06, boot *SE* = 0.33, 95% CI [−0.67, 0.66]. This means that the negative indirect effect of the disclosure on brand attitude via the understanding of selling intent disappears for children who do experience PSR with the YouTuber. The indexes of moderated mediation for ad recognition and understanding of persuasive intent were not significant. We did not find a significant moderated mediation of the disclosure for the children who did not correctly recall it (vs. no disclosure).

With regard to **advertised product desire** and **video attitude**, all indexes of moderated mediation were non-significant. This means that the indirect effect of the disclosure on advertised product desire via understanding of selling intent was not moderated by PSR. In addition, we found no support for any indirect effect of the disclosure on children’s attitude toward the video.

Altogether, this means that we found partial support for the moderated mediation proposed in H3a: The negative effect of one of the components of ad literacy (i.e., the understanding of selling intent) on brand attitude was negative and significant for children with low levels of PSR and was statistically non-significant for those with high levels of PSR. We found no support for H3b and H3c.

### Effects of No Disclosure vs. Disclosure

We also ran all analyses comparing the original two conditions – no disclosure vs. disclosure – without considering children’s disclosure recall. The results were the same to those discussed above: compared to no disclosure, the disclosure increased all ad literacy measures (see Appendix Table A1). In addition, we found the same significant mediation and moderated mediation effects. However, the mean differences and effects sizes were smaller, indicating that including the children who had not recalled the disclosure mitigated but did not nullify the effects of the disclosure.

## Discussion

This study provides new insights into the effects of disclosures on the activation of children’s advertising literacy in the context of influencer marketing in online videos. In addition, this study illuminates the effects of disclosures on children’s responses to the brand and the video itself. Finally, we provide evidence for the moderating role of children’s PSR with the influencer in disclosure effects.

Together this study leads to four main conclusions. First, we show that the disclosure used in this study is an effective means to inform children about the persuasive nature of a sponsored influencer video. More precisely, when children correctly remember it, the disclosure significantly enhanced children’s recognition of advertising, and their understanding of the selling and persuasive intent of the influencer video. Thus, it seems that the disclosure (if noticed and remembered) can trigger children’s knowledge about advertising, by explicitly alerting them to payment by the brand for advertising in the video.

These findings are in line with previous studies (De Jans et al., 2018a, b; De Pauw et al., 2018) that showed the effectiveness of disclosures in enhancing the children’s recognition of advertising. Our findings add to these studies that not only recognition of advertising, but also understanding the intent of the content can be activated by a disclosure. Understanding of intent has been found to be a crucial step in critical processing of advertising among children (Rozendaal et al., 2009). Therefore, this finding has important consequences for the empowerment of children to critically process influencer marketing.

Our findings are contrary to the conclusions of An and Stern (2011) and Panic et al. (2013), who found no effects of disclosures in advergames on children’s advertising literacy. These differences may be due to the medium that was studied: video/television programming versus advergames. The non-significant findings for advergames may be explained by the interactive nature of this medium. Perhaps playing an advergames requires so much resources that there is no cognitive capacity left to process the disclosure or to apply existing advertising literacy to the specific game (Mizerski et al., 2017). Based on previous studies (De Jans et al., 2018a, b) and our own study, we conclude that for (online) audiovisual sponsored content, a disclosure can activate various aspects of children’s advertising literacy.

Our second conclusion is that the various elements of advertising literacy play different roles in the persuasion process. On the one hand, the disclosure activates a cognitive process which leads to higher brand recall both directly and indirectly via recognition of advertising. This finding is in line with previous studies among adults and adolescents, that also showed that disclosures enhanced brand memory directly and indirectly via recognition of advertising (e.g., Boerman et al., 2012; Van Reijmersdal et al., 2017). As illustrated by eye tracking studies, disclosures can lead to more visual attention to the placement, which leads to better processing of the placement as indicated by enhanced brand memory (Boerman et al., 2014; Wojdynski and Evans, 2016; Guo et al., 2018). Interestingly, ad recognition did not affect brand attitude or advertised product desire. It seems that recognition of advertising triggers a cognitive process in which children’s attention to the content is enhanced leading to increased memory, but not to an affective process that influences evaluations of the brand.

On the other hand, the disclosure leads to a more affective process in which the understanding that the content is created to sell products leads to lower advertised product desire. Thus, via understanding of the selling intent of the influencer video, the disclosure seems to lead to resistance among children which is characterized by lower product desire. These effects can be explained by psychological reactance in response to restrictions of freedom (Brehm, 1966). If children understand that the video is created to sell them products, they may feel restricted in their freedom to choose for themselves which product to buy. To restore their freedom of choice, they seem to resist the persuasion attempt and respond with lower advertised product desire. It seems as if the children think, if this brand want to sell me something, I will not do it, and I do not want to eat fish sticks for dinner tonight (Knowles and Linn, 2004). This finding is in line with studies among children that also found negative effects of disclosures on purchase intention and advertised product desire (e.g., An and Stern, 2011; Panic et al., 2013; De Jans et al., 2018a). However, the majority of research on the relation between advertising literacy and persuasion failed to find effects on brand responses (see, Mizerski et al., 2017). These differences in effects may be explained by the type of advertising literacy and the type of persuasion that is involved. Our study revealed two seemingly dispersed processes: disclosures enhance cognitive brand effects via recognition of advertising, but not via understanding intent. At the same time disclosures decrease affective brand responses via understanding selling intent, but not via understanding persuasive intent or via recognition of advertising.

Our third conclusion is that the para-social relation between a child and an influencer is an important boundary condition for the effects of disclosures on brand attitudes. Our findings show that there is a divergent negative moderation effect of PSR (Holbert and Park, 2019): The understanding of selling intent evoked by the disclosure only leads to negative brand attitudes among children with medium to low levels of PSR with the influencer. For children who feel a strong PSR with the influencer, the understanding that the video is created to sell products does not lead to negative brand attitudes. For these children, their relationship with the influencer makes them less critical toward the sponsored content. They seem to be less resistant to what the influencer is telling them, although they realize that the intention is to sell these products. For children with lower PSR, their realization that the video is created to sell products, triggers resistance resulting in more negative attitude toward the brand. These findings converge with Dekker and Van Reijmersdal (2013) who showed that disclosures only had negative effects on brand responses among people who did not find the influencer (Oprah Winfrey) credible. Thus, relations with the influencer or perceptions of credibility may offset negative consequences of advertising literacy for the brand. Although the disclosure enhances advertising literacy, people who feel connected with the influencer or think highly of him or her do not feel the need to resist the persuasive attempt, probably because they trust the influencer (Lee and Watkins, 2016; Chen, 2018; Folkvord et al., 2019; Munnukka et al., 2019).

Fourth, our study showed that disclosures do not affect children’s attitude toward the video through advertising literacy. Even if a disclosure enhances children’s recognition of advertising in the video and their understanding that the video is created to sell products or to persuade, this does not have any consequences for their evaluation of the video itself. These findings are in line with the only previous study that included children’s evaluation of the content, that is an advergame (Van Reijmersdal et al., 2012). It seems that children use other criteria to evaluate the video than whether it is advertising or not, or whether it has an ulterior motive.

### Limitations and Suggestions for Future Research

Although this study provides new insights into the effects of disclosures on the activation of children’s advertising literacy, the persuasion process and boundary conditions of these effects, this study has some limitations. First, this study used a specific disclosure that included the brand name, payment by the brand, and the fact that there is advertising. Research among adults has shown that the formulation of the disclosure plays an important role in their effects on activating advertising literacy (Van Reijmersdal, 2016; Wojdynski and Evans, 2016). Future research is needed to see whether less explicit disclosures have the same effects on children’s advertising literacy activation.

Second, our study showed that children’s understanding of the selling intent of the video did play a role in the persuasion process, but that their understanding of the persuasive intent did not affect persuasion. Perhaps, children of this age are unable to fully grasp what it means if content has a persuasive intent, that is that is tries to manipulate thoughts and feelings, instead of concrete behaviors which is the case for understanding selling intent. If they do not understand the possible consequence of a persuasive intent, then it is also unlikely that this understanding results in resistance. The literature has shown that understanding persuasive intent develops later than understanding selling intent (John, 1999; Rozendaal et al., 2011; Lapierre, 2015). Although the disclosure activated children’s understanding of persuasive intent in our study, the children may still lack the ability to apply this knowledge (Rozendaal et al., 2011). Future research is needed to further understand how understanding selling intent and understanding persuasive intent are related to persuasion among children of between 8 and 12 years old.

Third, future research is needed to gain more insights into the role of PSRs in disclosure effects among younger and older children than the ones included here. Although previous studies have found evidence for PSRs among people of various ages (Folkvord et al., 2019; Munnukka et al., 2019) it remains unclear whether the strength of these relationships vary with age and whether age moderated the moderation effect of PSR in the relation between advertising literacy and persuasion.

Fourth, our study used one video by a specific male YouTuber for one branded product. Therefore, our findings may not be generalizable to other types of videos, YouTubers, or brands. The product was rather implicitly promoted in the video used in this study, however other videos – such as review or unboxing videos – may present a more nuanced picture of the pros and cons of a product. Similarly, the YouTuber in this study makes videos about his own life, which may make it easier for children to form PSRs with this YouTuber, compared to other YouTubers such as gamers who do not share information about their private life. In addition, our stimulus material included a low involvement product (fish sticks), but it remains uncertain whether a high involvement product or a product that has social value will be evaluated similarly after exposure to disclosures. Thus, future research is needed to show whether the disclosure and PSRs have the same effects for other types of videos, YouTubers, and products.

Fifth, the disclosure only had an effect for the children who noticed the disclosure and correctly remembered its content. Although this study is not the first to emphasize that disclosure recall is an important prerequisite for any disclosure effects (see Boerman et al., 2014, 2017; Van Reijmersdal et al., 2017), this study was the first to show that the disclosure effects mitigated, but not nullified, when disclosure recall was *not* taken into account. This provides evidence for the notion that, in other disclosure studies, the effects of a disclosure may even be stronger when disclosure recall is taken into account. Therefore, our study implies that future research should take the role of disclosure recall in disclosure effects among children into account.

### Theoretical and Practical Implications

Our findings have several implications for theory and practice. Theoretically, this study provides significant insights into the dispersed processes that underly disclosure effects on persuasion. Previous research among children showed mixed evidence on the relationship between advertising literacy and persuasion both with and without cues such as disclosures (see Mizerski et al., 2017). Our study shows that the relation between advertising literacy and persuasion may not be as straight forward as assumed so far. Effects seem to depend on the aspect of advertising literacy and the specific persuasive outcome that is investigated. We showed that ad recognition as evoked by a disclosure only affects brand memory (not brand attitude or product desire), whereas understanding selling intent did influence brand attitudes and advertised product desire (but not brand memory) and understanding persuasive intent did not affect any responses to the brand, product, and video. This calls for further refinement of theories that explain the link between advertising literacy and persuasion taking dispersed processes into account.

Furthermore, our study showed that the PSR that children experience with an influencer is an important boundary condition for effects of disclosures through advertising literacy to occur. This also adds theoretical refinement to the interpretation of previous studies. A lack of effects of advertising literacy on brand attitudes may be caused by children’s high levels of PSR. By showing for which children disclosures do and do not affect persuasion, our study provides theoretical insights into the impact of disclosure on children.

Practically, our study has implications for regulation, advertisers, and influencers. For regulation, our study implies that the disclosures used in this study *can* enhance the transparency of sponsored influencer videos among children aged 8–12. This means that disclosures can serve as an important tool to empower children in their understanding of the commercial nature of such videos. Our study also implies that disclosures can make children more resistant to persuasion by influencer marketing in online videos. Disclosures decreased advertised product desire for all children via understanding of selling intent, and for those children with low or medium levels of PSR disclosures also lowered their brand attitudes.

For advertisers, our study implies that disclosures can be beneficial for brand memory. In addition, for those children who score high on PSR with the influencer disclosures enhance transparency, without negative consequences for brand attitudes. Practically, advertisers should select influencers with audiences who experience strong PSR. However, advertisers should consider that disclosures do lower advertised product desire.

For influencers, our findings imply that disclosures have no consequences for children’s attitude toward the video itself. Thus, influencers can be transparent our sponsoring in their videos without risking to damage children’s evaluation of the video itself.

## Data Availability Statement

The datasets generated for this study are available on request to the corresponding author.

## Ethics Statement

The studies involving human participants were reviewed and approved by the University of Amsterdam, Department Persuasive Communication, ERB number 2017-PC-8594. Written informed consent to participate in this study was provided by the participants’ legal guardian/next of kin.

## Author Contributions

SB and ER designed the experiment and wrote the manuscript together. SB supervised the data collection and analyzed the data.

## Conflict of Interest

The authors declare that the research was conducted in the absence of any commercial or financial relationships that could be construed as a potential conflict of interest.

## Footnotes

1 To explore the effects of age more elaborately, we ran an additional MANOVA with the disclosure conditions and age (grouped: comparing 8–10 years old to 11–12 years old) as factors, and ad recognition, understanding of selling intent, and understanding persuasive intent as dependent variables. The results showed no significant main or interaction effect of the age groups on all three ad literacy measures (*p*’s > 0.127).

## Appendix

**TABLE A1:** Effect of disclosure (vs. no disclosure) on advertising literacy and brand, product, and video responses.

	No disclosure (*n* = 54)	Disclosure (*n* = 58)
Ad recognition	3.59^(1.28)a^	4.59^(1.32)b^
Understanding of selling intent	2.73^(1.16)a^	3.41^(1.20)b^
Understanding persuasive intent	3.35^(1.20)a^	3.94^(1.19)b^
Brand recall	55.6%^a^	79.3%^b^
Brand attitude	4.48^(1.11)a^	4.38^(1.43)a^
Advertised product desire	3.56^(1.75)a^	3.31^(1.88)a^
Video attitude	3.78^(0.97)a^	3.66^(0.89)a^

**^*a,b*^Means with a different superscript differ significantly at p < 0.05.**
